# The CRK2-CYC13 complex functions as an S-phase cyclin-dependent kinase to promote DNA replication in *Trypanosoma brucei*

**DOI:** 10.1186/s12915-021-00961-1

**Published:** 2021-02-11

**Authors:** Kyu Joon Lee, Ziyin Li

**Affiliations:** Department of Microbiology and Molecular Genetics, McGovern Medical School, University of Texas Health Science Center at Houston, Houston, TX 77030 USA

**Keywords:** *Trypanosoma brucei*, Mcm3, Sld5, Cdc45-Mcm2–7-GINS complex, Cdc2-related kinase, DNA replication

## Abstract

**Background:**

Faithful DNA replication is essential to maintain genomic stability in all living organisms, and the regulatory pathway for DNA replication initiation is conserved from yeast to humans. The evolutionarily ancient human parasite *Trypanosoma brucei*, however, lacks many of the conserved DNA replication factors and may employ unusual mechanisms for DNA replication. Neither the S-phase cyclin-dependent kinase (CDK) nor the regulatory pathway governing DNA replication has been previously identified in *T. brucei*.

**Results:**

Here we report that CRK2 (Cdc2-related kinase 2) complexes with CYC13 (Cyclin13) and functions as an S-phase CDK to promote DNA replication in *T. brucei*. We further show that CRK2 phosphorylates Mcm3, a subunit of the Mcm2–7 sub-complex of the Cdc45-Mcm2–7-GINS complex, and demonstrate that Mcm3 phosphorylation by CRK2 facilitates interaction with Sld5, a subunit of the GINS sub-complex of the Cdc45-Mcm2–7-GINS complex.

**Conclusions:**

These results identify the CRK2-CYC13 complex as an S-phase regulator in *T. brucei* and reveal its role in regulating DNA replication through promoting the assembly of the Cdc45-Mcm2–7-GINS complex.

**Supplementary Information:**

The online version contains supplementary material available at 10.1186/s12915-021-00961-1.

## Background

High fidelity DNA replication is crucial for maintaining genomic integrity in all living organisms. Eukaryotic organisms use a replication licensing system to ensure that the genome is replicated once, and only once, per cell cycle. In the G1 phase of the cell cycle, the origin recognition complex (ORC), which is composed of six closely related AAA-type ATPases named Orc1 to Orc6, occupies the replication origins and loads another AAA-type ATPase named Cdc6 onto the origins. Cdc6 further recruits Cdt1 and the Mcm2–7 complex onto the origins, thereby forming a pre-replicative complex [1, 2]. Initiation of DNA replication requires the phosphorylation of multiple DNA replication factors by Dbf4-dependent kinase (DDK) or Cdk7 and S-phase cyclin-dependent kinase (CDK) [3]. The DDK/Cdk7 phosphorylates several subunits of the Mcm2–7 complex and promotes interaction with Cdc45 [4]. S-phase CDK phosphorylates replication factors Sld2 and Sld3 [5, 6] to facilitate their binding to Dpb11 [7]. Sld2, Dpb11, the GINS complex composed of Sld5, Psf1, Psf2, and Psf3, and the leading-strand polymerase ε (Pol ε) form the pre-loading complex [8–11]. Sld3 interacts with Cdc45 and together they are recruited onto origins through interactions between Cdc45 and Mcm2–7 [4, 12, 13]. Interactions between Dpb11 and Sld3-Cdc45 help recruit the pre-loading complex onto origins [3]. Subsequently, Cdc6 (in budding yeast) or Cdt1 (in animals) is degraded, and Dpb11, Sld2, and Sld3 all dissociate from the replisome [3], leaving DNA polymerase and the CMG (Cdc45-Mcm2–7-GINS) complex at the replication origins. The CMG complex functions as a replicative helicase to unwind the duplex DNA for the DNA polymerase to replicate DNA [14–17].

*Trypanosoma brucei* is an early branching protozoan parasite [18] causing human sleeping sickness in sub-Saharan Africa and possesses numerous unusual biological features that might be exploited as drug targets. The regulation of DNA replication in *T. brucei*, although poorly understood, appears to possess both evolutionarily conserved and trypanosome-specific features [19–21]. *T. brucei* expresses a highly divergent ORC complex, of which only five subunits have been identified [22–24], and a conserved CMG complex [22], but its genome does not encode close homologs of Cdc6, Cdt1, and DDK/Cdk7 [25]. It appears that *T. brucei* may employ a control mechanism for DNA replication that is distinct from that in yeast and animals.

The S-phase CDK, a crucial DNA replication regulator that helps recruitment of the pre-loading complex to the replication origins [3], and its associated cyclin partner have not been discovered in *T. brucei*. The genome of *T. brucei* encodes eleven Cdc2-related kinases (CRK1–CRK4 and CRK6–CRK12) and eleven cyclins (CYC2–CYC12) [26], but it remains unknown which CRK-CYC pair functions as an S-phase CDK-cyclin complex to promote DNA replication. Here, we discover CRK2 as an S-phase CDK to complex with a new cyclin, CYC13, to promote DNA replication and S-phase progression in *T. brucei*. We also uncover a mechanistic role of CRK2 in DNA replication by demonstrating that CRK2 phosphorylates the Mcm2–7 complex subunit Mcm3 to facilitate the interaction of Mcm3 with the GINS complex subunit Sld5. These findings reveal a novel mechanism for CRK2-mediated DNA replication and highlight the unusual biology of cell cycle regulation in this early divergent eukaryote.

## Results

### CYC13 is the cyclin partner of CRK2 in *T. brucei*

**Fig. 1:**
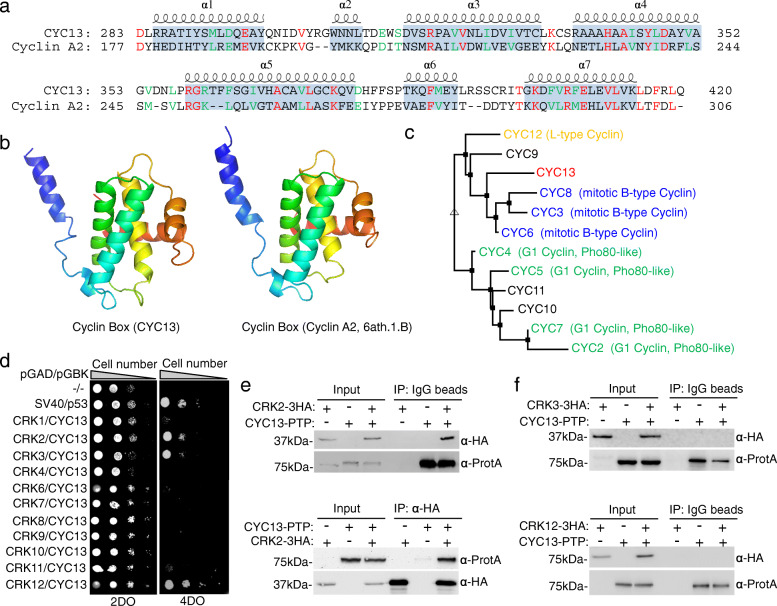
CYC13 is the cyclin partner of the CDK-related kinase CRK2 in *T. brucei*. **a** Sequence alignment of the cyclin-box domain of CYC13 and the human Cyclin A2. The α helices are based on the structure of the cyclin-box domain of Cyclin A2. Residues in red and in green are identical or conserved residues between CYC13 and Cyclin A2. **b**. Modeling of the cyclin-box domain of CYC13 by SWISS-MODEL using the template 6ath.1.B (cyclin-box domain of Cyclin A2). **c** Phylogenetic analysis of trypanosome cyclins, CYC2–CYC13. **d** Yeast two-hybrid assays to test the potential interaction between CYC13 and the 11 CDK-related kinases (CRKs). 2DO, double drop-out; 4DO, quadruple drop-out. **e**, **f** Co-immunoprecipitation to test the in vivo interaction between CYC13 and the three CRKs that interact with CYC13 in yeast two-hybrid. CYC13 was endogenously tagged with a PTP epitope, whereas CRK2, CRK3, and CRK12 were endogenously tagged with a triple HA epitope. CYC13-PTP alone and 3HA-tagged CRK2, CRK3, and CRK12 alone were included as negative controls

Hidden Markov Model (HMM) analysis identified 12 cyclin box-containing proteins in *T. brucei* shown in the superfamily HMM library and genome assignments server (http://supfam.org/SUPERFAMILY/cgi-bin/genome.cgi?sf=47954&listtype=sf&cgi_Tb=yes). Two of these proteins, encoded by Tb927.11.780 (or Tb11.03.0350) and Tb927.11.11500 (or Tb11.01.3350), have not been characterized previously. Tb927.11.11500 encodes a CDK-binding protein homologous to human CABLES protein [27], whereas the protein encoded by Tb927.11.780 contains a cyclin box-like domain (a.a. 272–420; *E*-value: 2.61e−16) and thus was named CYC13. The cyclin-box domain of CYC13 has an overall 19% sequence identity and a similar structure to the cyclin-box domain of human cyclin A2 (Fig. 1a, b), as predicted by structural modeling using SWISS-MODEL [28]. Cyclin A2 in humans activates both Cdk2 and Cdk1 to regulate S-phase and mitosis, respectively [29]. Sequence analyses of all trypanosome cyclins, CYC2–CYC13, showed that these cyclins do not share an overall sequence similarity (Additional file 1: Figure S1), because they belong to distinct types of cyclins. Phylogenetic analysis of these cyclins placed CYC13 to a distinct clade, to which the closest clade is the mitotic B-type cyclin clade (Fig. 1c).

To identify the CRK partner(s) of CYC13, we first carried out yeast two-hybrid assays to test its interaction with any of the eleven CRKs, and the results showed that CYC13 interacts with CRK2, CRK3, and CRK12 (Fig. 1d). Further, we carried out co-immunoprecipitation to test the in vivo interaction between CYC13 and the three CRKs. To this end, CYC13 was endogenously tagged with a PTP epitope in the trypanosome cells that express CRK2, CRK3, or CRK12 fused with a C-terminal triple HA epitope from their respective endogenous locus. Immunoprecipitation of CYC13-PTP was able to pull down CRK2-3HA, and immunoprecipitation of CRK2-3HA was able to pull down CYC13-PTP (Fig. 1e). However, immunoprecipitation of CYC13-PTP did not pull down CRK3-3HA and CRK12-3HA (Fig. 1f), and immunoprecipitation of CRK3-3HA or CRK12-3HA did not pull down CYC13-PTP (Additional file 1: Figure S2). These results identified CRK2 as the CDK partner of CYC13 in *T. brucei*.

### The CRK2-CYC13 complex is required for S-phase progression

**Fig. 2:**
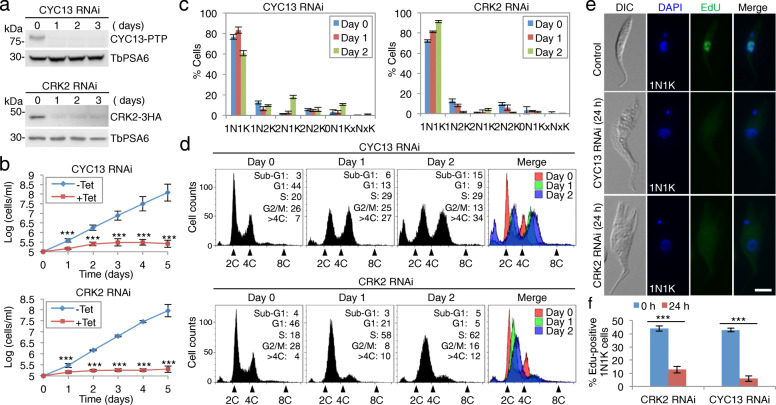
Knockdown of CYC13 and CRK2 by tetracycline-inducible RNAi caused defective S-phase progression. **a** Western blotting to detect CYC13-PTP and CRK2-3HA in their respective RNAi cells. TbPSA6 served as the loading control. **b** Cell growth curves of the CYC13 RNAi cell line and the CRK2 RNAi cell line. ***p < 0.001 (chi-square test). **c**. Effect of CYC13 and CRK2 depletion on cell cycle progression. Cells with different numbers of nucleus (N) and kinetoplast (K) before and after RNAi induction were counted. A total of 100 cells were counted for each time point, and error bars indicated S.D. from three independent experiments (*n* = 3). **d** Flow cytometry analysis of CYC13 RNAi cells and CRK2 RNAi cells. Insets show the percentage of cells at different cell cycle stages. **e** EdU incorporation assay in non-induced control, CYC13 RNAi, and CRK2 RNAi cells. Scale bar: 5 μm. **f** Quantitation of EdU-positive 1N1K cells in non-induced control, CYC13 RNAi-induced cells, and CRK2 RNAi-induced cells. Error bars indicate S.D. from three independent experiments (*n* = 3). ***p < 0.001 (chi-square test)

We carried out RNAi to investigate the function of the CRK2-CYC13 complex in the procyclic form of *T. brucei*. To assess the efficiency of RNAi, we epitope-tagged CYC13 and CRK2 from their respective endogenous locus in their respective RNAi cell line for western blotting. Induction of CYC13 RNAi and CRK2 RNAi each resulted in the reduction of their protein levels by > 90% after 1 day (Fig. 2a), indicating efficient depletion of the target protein by RNAi. Depletion of CYC13 and CRK2 each caused severe growth defects (Fig. 2b), suggesting that both proteins are essential for cell proliferation. We further analyzed the potential effects of CYC13 RNAi and CRK2 RNAi on cell cycle progression by counting the cells with different numbers of kinetoplasts and nuclei. *T. brucei* cells at different cell cycle stages can be readily distinguished by the numbers of kinetoplasts and nuclei. Cells at the G1 phase and early S phase of the cell cycle contain one nucleus and one kinetoplast (1N1K), cells at late S phase to early anaphase contain one nucleus and two kinetoplasts (1N2K), and cells at late anaphase to cytokinesis contain two nuclei and two kinetoplasts (2N2K). Knockdown of CYC13 caused an initial increase of 1N1K cells at 24 h and subsequently a decrease of 1N1K and an emergence of two abnormal cell types, the 2N1K cells (~ 18%) and 0N1K cells (~ 11%), at 48 h (Fig. 2c). Unlike knockdown of CYC13, however, knockdown of CRK2 caused a gradual increase of 1N1K cells from ~ 76 to ~ 91% after RNAi induction for 48 h, without a significant accumulation of abnormal cell types, the 2N1K cells and the 0N1K cells (Fig. 2c).

To further characterize the cell cycle defects of the two RNAi cell lines, flow cytometry was carried out. Knockdown of CYC13 caused a decrease of G1 cells by 35% and G2/M cells by 13%, and a corresponding increase of S-phase cells by 9%, cells with DNA content greater than 4C by 27%, and anucleate (sub-G1) cells by 12% at 48 h of RNAi (Fig. 2d). The emergence of cells with DNA content between 4C and 8C suggests that the bi-nucleated cells were undergoing the next round of DNA replication before cell division, and the accumulation of anucleate cells indicates aberrant cytokinesis of some of the bi-nucleated cells, which produced 2N1K and 0N1K cells. This profound effect of CYC13 knockdown on the cell cycle suggests that CYC13 may play multiple roles at different cell cycle stages, including S phase. Knockdown of CRK2 caused a decrease of G1 cells by 41% and G2/M cells by 12%, and a corresponding increase of S-phase cells by 44% and cells with DNA content greater than 4C by 8%, but no significant increase of anucleate cells (Fig. 2d). These results suggest that CRK2 plays a role in S-phase progression.

We next carried out EdU incorporation assay to examine the effect of CYC13 RNAi and CRK2 RNAi on DNA replication. Non-induced control and RNAi-induced cells were incubated with EdU, and the incorporation of EdU into DNA was monitored by fluorescence microscopy and by counting the number of EdU-positive 1N1K cells (Fig. 2e, f). In the non-induced control cells, ~ 43% of the 1N1K cells were EdU-positive (Fig. 2e, f), indicating that these cells were undergoing DNA replication. In the RNAi-induced cells, ~ 13% and ~ 6% of the 1N1K cells were EdU-positive for CRK2 RNAi and CYC13 RNAi, respectively (Fig. 2e, f), suggesting that the majority of the RNAi-induced cells failed to incorporate EdU into DNA. These results further demonstrated that knockdown of CYC13 and CRK2 impaired DNA replication.

**Fig. 3:**
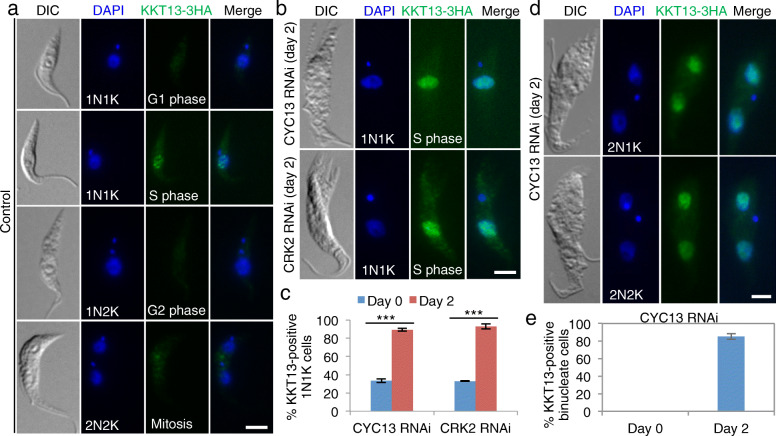
Knockdown of CYC13 and CRK2 by tetracycline-inducible RNAi increased S-phase cells. **a** KKT13 as an S-phase marker in *T. brucei*. Shown is the localization of KKT13, which was tagged with a triple HA epitope from its endogenous locus in the CYC13 RNAi cell line and the CRK2 RNAi cell line, in non-induced control cells. Scale bar: 5 μm. **b** KKT13 expression in CYC13 RNAi-induced and CRK2 RNAi-induced cells. KKT13 was detected with FITC-conjugated anti-HA antibody. Scale bar: 5 μm. **c** Quantitation of KKT13-positive 1N1K cells before and after CYC13 RNAi and CRK2 RNAi induction. A total of 100 cells were counted for each cell line and each time point, and error bars indicated S.D. from three independent experiments (*n* = 3). ***p < 0.001 (chi-square test). **d** KKT13-3HA was detected in the two nuclei of CYC13-depleted bi-nucleated (2N1K and 2N2K) cells. Scale bar: 5 μm. **e** Quantitation of CYC13-positive bi-nucleate cells in non-induced control and CYC13 RNAi-induced cells. Error bars indicate S.D. from three independent experiments (*n* = 3)

Finally, we carried out immunofluorescence microscopy using KKT13 as an S-phase marker to examine the effect of CYC13 RNAi and CRK2 RNAi on S-phase progression. KKT13 is a kinetochore protein and is only expressed during the S phase of the cell cycle in *T. brucei* [30], and thus it can serve as an S-phase marker to distinguish the 1N1K cells that are at either G1 phase or S phase. Immunostaining of KKT13 tagged with a C-terminal triple HA epitope from its endogenous locus was performed (Fig. 3), and the intensity of KKT13 fluorescence intensity was quantitated (Additional file 1: Figure S3), enabling us to distinguish between KKT13 fluorescence signal and the background signal for unambiguous counting of KKT13-positive cells. The results showed that the percentage of S-phase cells among the 1N1K cell population was increased by ~ 66% and ~ 70% after CYC13 knockdown and CRK2 knockdown, respectively (Fig. 3a–c). Since flow cytometry showed that the bi-nucleated cells of the CYC13 RNAi-induced cell population had entered the S phase of the next cell cycle (Fig. 2d), we examined KKT13 expression in the bi-nucleated cells (2N2K cells from the control cell population and 2N2K and 2N1K cells from the CYC13 RNAi-induced cell population). We found that the KKT13-positive bi-nucleated cells increased to ~ 85% after CYC13 RNAi induction for 48 h (Fig. 3d, e), confirming that the bi-nucleated cells were indeed arrested at the next round of S phase. All together, these results suggest that the CRK2-CYC13 complex plays an essential role in promoting S-phase progression.

### The Cdc45-Mcm2–7-GINS complex subunit Mcm3 is a substrate of CRK2

**Fig. 4:**
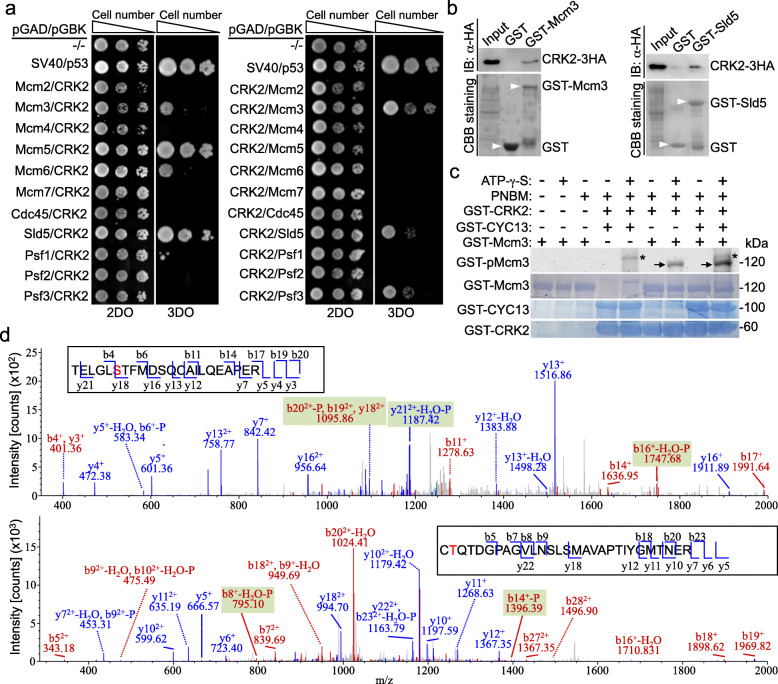
Mcm3 is a substrate of CRK2. **a** Yeast two-hybrid assays to test the potential interaction between CRK2 and the subunits of the CMG complex. 2DO, double drop-out; 3DO, triple drop-out. **b** GST pull-down to test the interaction between CRK2 and the CMG subunits Mcm3 and Sld5. GST-Mcm3 and GST-Sld5 were expressed and purified from bacteria, and CRK2-3HA was expressed in *T. brucei*. GST alone was used as the negative control. CBB, coomassie brilliant blue. **c** In vitro kinase assay of Mcm3 phosphorylation by CRK2 in the presence and absence of CYC13. GST-Mcm3, GST-CRK2, and GST-CYC13 were expressed and purified from *E. coli*. Arrows indicate phosphorylated GST-Mcm3 (GST-pMcm3), which was detected by the anti-ThioP antibody. GST-fused Mcm3, CRK2, and CYC13 were stained with coomassie brilliant blue. The asterisks indicate a non-specific band detected by the anti-ThioP antibody in the reactions to which purified GST-CYC13 was added. **d** MS/MS spectrum of Ser-213 (upper panel) and Thr-310 (lower panel) phosphopeptides identified from CRK2-phosphorylated GST-Mcm3. In vitro phosphorylated recombinant GST-Mcm3 was analyzed by LC-MS/MS. The phosphorylated Ser-213 and Thr-310 residues are shown in red

DNA replication in eukaryotes requires a cohort of DNA replication factors, including the CMG complex that functions as a replicative helicase to unwind the DNA duplex during DNA replication in the S phase of the cell cycle [17]. Since the CRK2-CYC13 complex is required for DNA replication (Figs. 2 and 3), we asked whether CRK2 regulates the CMG complex by interacting and phosphorylating any of the CMG subunits. To test this possibility, we first carried out yeast two-hybrid assays to screen for the CRK2-interacting CMG subunit(s). CRK2 was fused with either the Gal4 DNA-binding domain (BD) or Gal4 activation domain (AD) and paired with each of the eleven CMG complex subunits that were fused with the AD or the BD (Fig. 4a). When fused with the BD, CRK2 interacted with Mcm3, Mcm5, Mcm6, and Sld5 (Fig. 4a, left panel), and when fused with the AD, CRK2 interacted with Mcm3, Sld5, and Psf3 (Fig. 4a, right panel). Collectively, both experiments identified Mcm3 and Sld5 as the interacting partners of CRK2. GST pull-down further confirmed that Mcm3 and Sld5 interacted with CRK2 in vitro (Fig. 4b).

To test whether Mcm3 and Sld5 are substrates of CRK2, in vitro kinase assay was performed with recombinant GST-fused proteins, GST-Mcm3, GST-Sld5, GST-CRK2, and GST-CYC13, all of which were expressed and purified from bacteria, using the non-radioactive thiophosphorylation method [31]. This method involves the use of the anti-ThioP antibody to detect thiophosphorylated proteins [31]. The in vitro kinase assay showed that CRK2 alone was able to phosphorylate Mcm3, and the addition of CYC13 increased the levels of phosphorylated Mcm3 (Fig. 4c), demonstrating that CYC13 promotes the kinase activity of CRK2. Further, to identify the phosphosites, mass spectrometry was carried out after in vitro kinase assay. A total of 1097 peptides, which were represented by 16 unique peptides and covered ~ 76% of the entire protein, were identified for Sld5, and a total of 3997 peptides, which were represented by 91 unique peptides and covered ~ 88% of the entire protein, were identified for Mcm3 (Additional file 1: Figure S4). Two phospho-peptides, which contained phosphorylated Ser-213 and Thr-310, respectively (Fig. 4d), were identified for Mcm3, but not for Sld5, indicating that Mcm3 is a substrate of CRK2.

### Phosphorylation of Mcm3 at Thr-310 is essential for cell proliferation

**Fig. 5:**
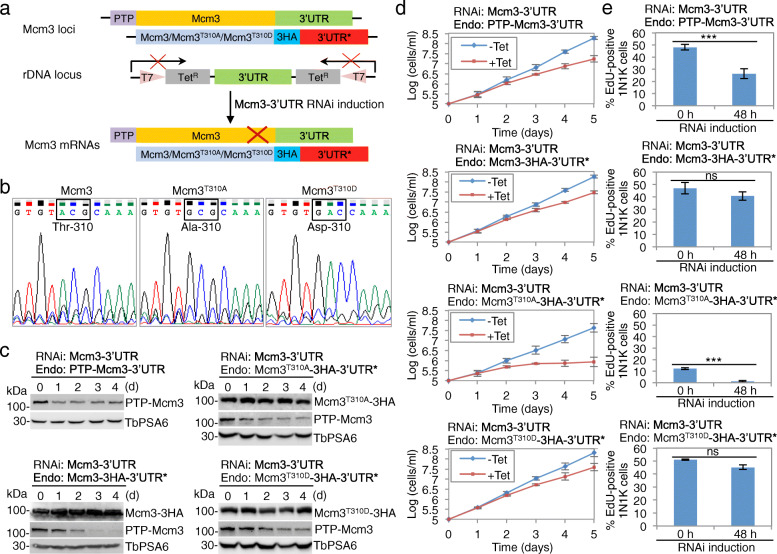
CRK2 phosphorylation of Thr-310 in Mcm3 is essential for cell proliferation. **a** Schematic flowchart illustrating the Mcm3-3′UTR RNAi complementation strategy. The 3′UTR* in the red box indicates RNAi-resistant 3′UTR sequence from a different gene. **b** Analysis of the sequences flanking the codon encoding wild-type and mutated Thr-310 residue in the replaced *Mcm3* locus. **c** Western blotting to monitor the knockdown of Mcm3, which was tagged with a PTP epitope, and the expression of RNAi-resistant wild-type and mutant Mcm3 proteins, which was tagged with a triple HA epitope. in non-induced control and Mcm3-3′UTR RNAi-induced cells. PTP-Mcm3 was detected by anti-protein A antibody, whereas Mcm3-3HA, Mcm3^T310A^-3HA, and Mcm3^T310D^-3HA were detected by anti-HA antibody. TbPSA6 served as a loading control. **d** Effect of CRK2-mediated phosphorylation of Thr-310 in Mcm3 on cell proliferation. Shown are growth curves of Mcm3-3′UTR RNAi cell line and Mcm3-3′UTR RNAi complementation cell lines. **e** Effect of Mcm3 knockdown and Mcm3 RNAi complementation by wild-type and mutant Mcm3 on EdU incorporation. Shown is the quantitation of EdU-positive 1N1K cells before and after tetracycline induction for 48 h. Error bars indicate S.D. from three independent experiments (*n* = 3). ***p < 0.001; ns, no significance (chi-square test)

To test whether phosphorylation of Ser-213 and Thr-310 in Mcm3 is essential for Mcm3 function, we carried out a functional complementation experiment (Fig. 5a). We first generated a Mcm3-3′UTR RNAi cell line by inserting an RNAi construct that targets the 3′UTR of *Mcm3* gene into the rDNA locus. Subsequently, we inserted the PTP epitope-encoding sequence at the 5′-end of one allele of *Mcm3* gene to express a PTP-Mcm3 fusion protein for monitoring RNAi efficiency. Finally, we replaced the other allele of *Mcm3* gene with the sequence that encodes either wild-type or mutant Mcm3 proteins fused with a C-terminal triple HA epitope followed by a different 3′UTR sequence that is resistant to Mcm3-3′UTR RNAi. When Mcm3-3′UTR RNAi is induced with tetracycline, the PTP-Mcm3 mRNA, but not the mRNA of 3HA-tagged wild-type and mutant Mcm3, will be knocked down (Fig. 5a), thus allowing to assess the effect of CRK2 phosphorylation on Mcm3 function. Epitope tagging of Mcm3 with either the triple HA epitope or the PTP epitope did not affect cell growth (Additional file 1: Figure S5). Using this approach, we attempted to generate the complementation cell lines for Ser-213 and Thr-310 and their respective mutants. However, we failed to replace Mcm3 with the Ser-213 phospho-deficient mutant (Mcm3^S213A^) and the Ser-213 phospho-mimic mutant (Mcm3^S213D^) despite numerous tries, indicating that mutation of Ser-213 caused dominant-negative effects and killed cells. Thus, it was impossible to assess the contribution of Ser-213 phosphorylation to Mcm3 function using this approach.

Replacement of Mcm3 with the Thr-310 phospho-deficient mutant (Mcm3^T310A^) and the Thr-310 phospho-mimic mutant (Mcm3^T310D^) was successful and was confirmed by sequencing of the PCR fragment amplified from the replaced Mcm3 allele (Fig. 5b). Western blotting showed that the N-terminal PTP-tagged Mcm3, but not the C-terminal 3HA-tagged Mcm3 and its mutants, was knocked down by Mcm3-3′UTR RNAi (Fig. 5c), confirming that RNAi of Mcm3 by targeting Mcm3-3′UTR was specific towards PTP-Mcm3. Induction of Mcm3 RNAi through targeting its 3′UTR caused partial reduction of Mcm3 protein level (Fig. 5c), and caused a moderate growth defect, resulting in a ~ 11-fold growth reduction after 5 days (Fig. 5d). EdU incorporation assay showed that induction of Mcm3-3′UTR RNAi for 48 h caused a reduction of the EdU-positive 1N1K cells from ~ 48 to ~ 26% (Fig. 5e). When one allele of Mcm3 was replaced with the RNAi-resistant wild-type Mcm3 and then RNAi was induced, PTP-Mcm3 was gradually knocked down and Mcm3-3HA was not affected (Fig. 5c), but cell growth was only partly restored, resulting in a ~6-fold growth reduction after 5 days (Fig. 5d). EdU incorporation assay showed that EdU-positive 1N1K cells were reduced from ~ 47 to ~ 41% after RNAi induction for 48 h (Fig. 5e). The lack of full rescue of the growth defect is likely due to the fact that only one allele of the *Mcm3* gene expressed Mcm3 protein after RNAi induction and that Mcm3 protein level is so tightly controlled that a slight or moderate modulation of its level impacts cell growth. As a support of the notion that Mcm3 protein level is stringently regulated, ectopic overexpression of Mcm3 in the 29–13 cell line caused a severe growth defect, resulting in a ~ 23-fold growth reduction after 5 days (Additional file 1: Figure S6).

When one allele of Mcm3 was replaced with the RNAi-resistant Mcm3^T310A^ mutant, the cell line was not growing as well as other cell lines even without RNAi induction (Fig. 5d), suggesting that expression of Mcm3^T310A^ might cause dominant-negative effects but allowed slower cell proliferation. This notion was supported by the observed severe growth defects caused by ectopic overexpression of Mcm3^T310A^ in the 29–13 cell line, which resulted in a ~ 400-fold growth reduction after 5 days (Figure S6). When Mcm3-3′UTR RNAi was induced, PTP-Mcm3 was gradually depleted and Mcm3^T310A^-3HA was unaffected (Fig. 5c), but cell growth was inhibited, resulting in a ~ 48-fold growth reduction after 5 days (Fig. 5d), indicating that the Mcm3^T310A^ mutant failed to restore cell proliferation. EdU incorporation assay showed that without RNAi induction the EdU-positive 1N1K cells only constituted ~ 12% of the 1N1K population, and after RNAi induction for 48 h EdU-positive 1N1K cells were reduced to ~ 1% of the total 1N1K population (Fig. 5e).

When one allele of Mcm3 was replaced with the RNAi-resistant Mcm3^T310D^ mutant and then RNAi was induced, PTP-Mcm3 was knocked down and Mcm3^T310D^-3HA was unaffected (Fig. 5c), and cell growth was partially restored, resulting in a ~ 5-fold growth reduction after 5 days (Fig. 5d), similar to the wild-type Mcm3 complementation cell line (Fig. 5d). EdU incorporation assay showed that the EdU-positive 1N1K cells were reduced from ~ 51 to ~ 45% after RNAi induction for 48 h (Fig. 5e). When Mcm3^T310D^ was ectopically overexpressed in the 29–13 cell line, it caused a moderate growth defect, resulting in a ~ 14-fold growth reduction after 5 days (Figure S6). Altogether, these results suggest that Thr-310 phosphorylation is essential for Mcm3 function in DNA replication.

### Phosphorylation of Mcm3 at Thr-310 is required for its interaction with Sld5

**Fig. 6:**
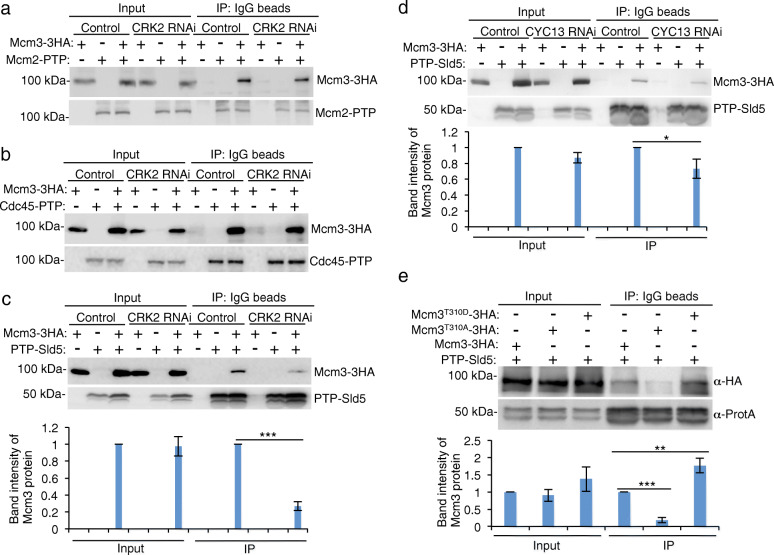
Phosphorylation of Thr-310 in Mcm3 by CRK2 promotes its interaction with Sld5. **a** Effect of CRK2 RNAi on Mcm2-Mcm3 interaction. Shown is the co-immunoprecipitation of Mcm2-PTP and Mcm3-3HA from control and CRK2 RNAi cells. Mcm2-PTP alone and Mcm3–3HA alone were included as negative controls. **b** Effect of CRK2 RNAi on Cdc45-Mcm3 interaction. Shown is the co-immunoprecipitation of Cdc45-PTP and Mcm3-3HA from control and CRK2 RNAi cells. Cdc45-PTP alone and Mcm3–3HA alone were included as negative controls. **c** Effect of CRK2 RNAi on Sld5-Mcm3 interaction. Shown is the co-immunoprecipitation of PTP-Sld5 and Mcm3-3HA from control and CRK2 RNAi cells. PTP-Sld5 alone and Mcm3-3HA alone were included as negative controls. The histogram below the western blots is the quantitation of Mcm3 band intensity, which was normalized against that of Sld5 from three independent experiments (*n* = 3). Error bars indicated S.D. ***p < 0.001 (chi-square test). The faster migrating band in the blot of PTP-Sld5 is a degradation product of PTP-Sld5. **d** Effect of CYC13 RNAi on Sld5-Mcm3 interaction. Shown is the co-immunoprecipitation of PTP-Sld5 and Mcm3-3HA from control and CYC13 RNAi cells. PTP-Sld5 alone and Mcm3-3HA alone were included as negative controls. The histogram below the western blots is the quantitation of Mcm3 band intensity, which was normalized against that of Sld5 from three independent experiments (*n* = 3). Error bars indicated S.D. *p < 0.05 (chi-square test). **e** Effect of Mcm3 Thr-310 phosphorylation on its interaction with Sld5. Shown is the co-immunoprecipitation of PTP-tagged Sld5 and 3HA-tagged wild-type and mutant Mcm3 proteins from *T. brucei*. The histogram below the western blots is the quantitation of the band intensity of wild-type and mutant Mcm3, which was normalized against that of Sld5 from three independent experiments (*n* = 3). Error bars indicated S.D. **p < 0.01; ***p < 0.001 (chi-square test)

Having established the functional essentiality of Mcm3 Thr-310 phosphorylation (Fig. 5), we explored the underlying mechanisms by investigating the potential effect of Mcm3 phosphorylation on its interaction with other subunits of the CMG complex. We chose the subunit protein Mcm2 from the Mcm2–7 sub-complex, the subunit protein Sld5 from the GINS sub-complex, and Cdc45 to test their interaction with Mcm3 in CRK2 RNAi cells. We co-expressed Mcm3-3HA with Mcm2-PTP, PTP-Sld5, or Cdc45-PTP in cells harboring the CRK2 RNAi construct, and then performed co-immunoprecipitation to test the interactions in control and CRK2 RNAi cells. We found that immunoprecipitation of Mcm2-PTP was able to pull down Mcm3-3HA and the interaction between them was not affected by CRK2 knockdown (Fig. 6a). Similarly, co-immunoprecipitation of Cdc45-PTP and Mcm3-3HA was also unaffected by CRK2 knockdown (Fig. 6b). However, co-immunoprecipitaiton of PTP-Sld5 and Mcm3-3HA was impaired by CRK2 knockdown (Fig. 6c). CYC13 knockdown also affected the interaction between Mcm3 and Sld5 (Fig. 6d), albeit the effect was weaker than that caused by CRK2 knockdown, likely because CRK2 kinase activity was only partially reduced in the absence of CYC13 (Fig. 4c). Further, to test whether phosphorylation of Thr-310 affects the interaction between Mcm3 and Sld5, we co-expressed PTP-Sld5 and 3HA-tagged wild-type Mcm3, phospho-deficient Mcm3 (Mcm3^T310A^), and phospho-mimic Mcm3 (Mcm3^T310D^), and then performed co-immunoprecipitation. The results showed that immunoprecipitation of Sld5 pulled down significantly less amounts of Mcm3^T310A^ protein, but significantly more amounts of Mcm3^T310D^ protein than wild-type Mcm3 (Fig. 6e). Altogether, these results suggest that phosphorylation of Thr-310 in Mcm3 by CRK2 is required for Mcm3 interaction with the GINS subunit protein Sld5, but not Cdc45 and the Mcm2–7 complex subunit protein Mcm2.

## Discussion

Tremendous efforts have been undertaken in the past to understand the functions of the expanded repertoire of cyclins and CRKs in *T. brucei* and to dissect the CRK-regulated cellular pathways in *T. brucei* [32–46], but the S-phase cyclin-CRK pair had been overlooked, making the understanding of the CDK-mediated regulation of DNA replication in *T. brucei* impossible. *T. brucei* possesses a unique molecular composition of the DNA replication initiation machinery [22–24, 47] and lacks several key regulators of DNA replication [19, 48], suggesting distinct mechanisms and regulatory pathways for DNA replication. With the discovery of the CYC13-CRK2 pair as a regulator of S-phase progression in *T. brucei*, we are now able to delineate the signaling cascade controlling DNA replication in this early branching eukaryote, which might provide novel insights into the understanding of the evolution of the DNA replication machinery in eukaryotes.

Flow cytometry analysis of CRK2 RNAi cells detected a major S-phase peak (Fig. 2d), indicating the arrest of cells at S phase. Since RNAi induction was initiated in asynchronous cells containing ~ 28% of the G2/M-phase cells (Fig. 2d), it suggests that these G2/M cells were able to divide, but the daughter cells were then arrested in the S phase of the next cell cycle. Hence, knockdown of CRK2 did not inhibit G2/M progression and cytokinesis. Flow cytometry analysis of CYC13 knockdown cells, however, detected an S-phase peak, a post-G2/M peak, and a smaller sub-G1 peak (Fig. 2d). We interpreted these observations as follows. Upon CYC13 RNAi induction, G1 cells progressed and then were arrested in S phase, producing uni-nucleated cells with DNA content greater than 2C but less than 4C (S-phase peak). However, G2/M cells re-initiated DNA replication without undergoing cytokinesis and then were arrested in the S phase of the next cell cycle, thus generating bi-nucleated cells with DNA content greater than 4C (post-G2/M peak). The anucleated cells (sub-G1 peak), together with the 2N1K cells (Fig. 2c), were generated through aberrant division of some of the bi-nucleated cells. Thus, CYC13 plays one role through activating CRK2 to promote S-phase progression and another role through activation of an unidentified CRK to promote cytokinesis. This unidentified CRK might be among the remaining eight CRKs that showed no interaction with CYC13 in yeast two-hybrid assays, but it may interact with CYC13 in vivo in trypanosomes. This subject is out of the scope of the current work and was not pursued further.

The identification of Mcm3 as a substrate of CRK2 (Fig. 4) and the finding that Mcm3 phosphorylation promotes Mcm3-Sld5 interaction (Fig. 6) provided the evidence to support the role of CRK2 in regulating S-phase progression. Mcm3 is an essential subunit of the CMG complex, the replicative helicase required for DNA replication initiation in eukaryotes [17]. *T. brucei* has a conserved CMG complex [22], but lacks the homolog of the Dbf4-dependent kinase (DDK), the major protein kinase responsible for Mcm2–7 phosphorylation [3]. DDK phosphorylates multiple Mcm proteins, such as Mcm2, Mcm4, and Mcm6 [4, 12, 49, 50], and phosphorylation of Mcm4 promotes the loading of Cdc45 to the Mcm2**–**7 complex [4]. CDK also phosphorylates multiple Mcm proteins, and phosphorylation of them plays distinct functions [51–55]. In human cell lines, Cdk1 phosphorylates Mcm3 at multiple sites and phosphorylation of Ser-112 by Cdk1 promotes Mcm3 incorporation into the Mcm2**–**7 complex [53]. Phosphorylation of Thr-722 in Mcm3 by Cdk2 promotes its association with chromatin [56]. However, in *T. brucei*, phosphorylation of Mcm3 at Thr-310 likely does not affect the formation of the Mcm2-Mcm3-Mcm5 sub-complex, but compromises the interaction between Mcm3 and Sld5 (Fig. 6), indicating the disruption of the interaction between Mcm2**–**7 and GINS within the CMG complex. It is possible that phosphorylation of different sites in Mcm3 might play distinct roles for Mcm3 function.

**Fig. 7:**
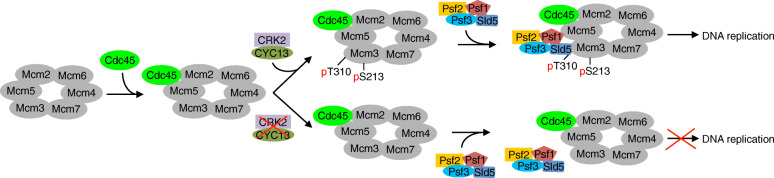
Model of CRK2-mediated phosphorylation of Mcm3 and its effect on DNA replication. Prior to the initiation of DNA replication, the Mcm2–7 complex was first loaded onto chromosomes, followed by the loading of Cdc45, likely through interactions with Mcm2 and Mcm5. Subsequently, CRK2 phosphorylates Mcm3 at Thr-310 and Ser-213, and phosphorylation of Thr-310 promotes the recruitment of the GINS complex to the Mcm2–7 complex, leading to the formation of the CMG complex and then the initiation of DNA replication. When CRK2 is deficient, the GINS complex is not recruited, and the CMG complex is not formed, thereby preventing the initiation of DNA replication

The CRK2 phosphosite on Mcm3, Thr-310, resides in the N-terminal intrinsically disordered region that is ~ 100 a.a. away from the Walker A motif of the AAA^+^ ATPase domain, indicating that this phosphorylation is unlikely to impact its ATPase activity. The Mcm4-Mcm6-Mcm7 sub-complex has helicase activity, and the Mcm2-Mcm3-Mcm5 sub-complex may play regulatory roles [57]. In *T. brucei*, only Mcm4, but not other Mcm proteins, possesses in vitro helicase activity [22]. Within the CMG complex, Cdc45 interacts with Mcm2, and the GINS sub-complex interacts with Mcm3 and Mcm5 [58]. Therefore, our finding that Mcm3 interacts with the Sld5 subunit of the GINS sub-complex in *T. brucei* (Fig. 6) suggests that *T. brucei* CMG complex has similar subunit arrangements as in other eukaryotes. Based on the well-established order of loading of Mcm2**–**7, Cdc45, and GINS to the replication origins and the known protein-protein interactions among the CMG subunits [22, 59], we propose the following model to depict the order of assembly of the CMG complex and its regulation by CRK2 in *T. brucei.* Mcm2**–**7 is among the first set of DNA replication regulators to be loaded onto the replication origins. This is followed by the loading of Cdc45 onto the vicinity of Mcm2 and Mcm5 through a DDK-independent mechanism and, finally, after phosphorylation of Thr-310 in Mcm3 by CRK2, the GINS sub-complex is loaded onto the vicinity of Mcm3 and Mcm5 to form the CMG complex, thus leading to the initiation of DNA replication (Fig. 7). When CRK2 is knocked down, Mcm3 is not phosphorylated and the GINS sub-complex is not loaded onto the vicinity of Cdc45 and Mcm2**–**7; therefore, assembly of the CMG complex is impaired and DNA replication is inhibited (Fig. 7). In this regard, CRK2 plays an essential role in DNA replication by facilitating the assembly of the CMG complex through promoting the interaction between Mcm3 and Sld5.

## Conclusions

We have identified CYC13-CRK2 pair as an S-phase cyclin-CDK in the early branching trypanosomes, and we provided evidence to show that CYC13-CRK2 promotes DNA replication initiation and S-phase progression. CYC13-CRK2 phosphorylates Mcm3, a subunit of the Mcm2–7 complex, and this phosphorylation promotes the interaction of Mcm3 with Sld5, a subunit of the GINS complex, thereby facilitating the assembly of the Cdc45-Mcm2–7-GINS complex. 

## Methods

### Yeast two-hybrid assay

Yeast two-hybrid assays were performed according to our published procedures [22]. Briefly, the full-length coding sequences of CYC13, the eleven CRKs, and the eleven subunits of the CMG complex were each cloned into the pGAD vector for expression of Gal4 activation domain fusion proteins (prey) or into the pGBK vector for expression of Gal4 DNA-binding domain fusion proteins (bait). Yeast strain AH109 (mating type a) and Y187 (mating type α) were transformed with the prey and the bait plasmids, respectively. Yeast mating was carried out by mixing the AH109 and Y187 strains in YPDA medium and incubating on the SD-Leu-Trp plates at 30 °C for 24 h. To test the interaction between prey and bait proteins, each combination stain was spotted in three 10-fold serial dilutions onto SD-Leu-Trp (2 drop-outs or 2DO), SD-Leu-Trp-His-Ade (3 drop-outs or 3DO), or SD-Leu-Trp-His-Ade (4 drop-outs or 4DO) plates, and then incubated at 30 °C for up to 3 days. Growth of yeast on the 3DO and 4DO plates indicates interaction between the prey and the bait proteins. The empty vectors of pGAD and pGBK were used as the negative control, and the pair of SV40-p53 was used as the positive control.

### Trypanosome cell culture and tetracycline-inducible RNA interference

The procyclic form of *T. brucei* strain Lister 427, which was used for epitope tagging of proteins, was cultured in SDM-79 medium supplemented with 10% heat-inactivated fetal bovine serum (FBS, Sigma-Aldrich) at 27 °C. The procyclic 29–13 cell line [60], which expresses T7 RNA polymerase and tetracycline repressor for inducible RNAi and overexpression of genes, was cultured in SDM-79 supplemented with 10% heat-inactivated FBS, 15 μg/ml G418, and 50 μg/ml hygromycin at 27 °C.

To generate the RNAi cell line for CYC13 and CRK2, the full-length coding sequence of CYC13 and a 417-bp DNA fragment (nucleotides 609–1025) from the coding region of CRK2 were each amplified by PCR and then cloned into the stem-loop RNAi vector, pSL [61], to generate the pSL-CYC13 and pSL-CRK2 constructs, respectively. The RNAi plasmid was linearized by NotI digestion and transfected into the 29–13 strain by electroporation. Transfectants were selected with 2.5 μg/ml phleomycin and cloned by limited dilution in a 96-well plate containing SDM-79 medium supplemented with 20% heat-inactivated FBS and appropriate antibiotics. RNAi was induced by incubating the cells with 1.0 μg/ml tetracycline.

### Ectopic overexpression of Mcm3 and its mutants

The full-length coding sequence of Mcm3 and its Thr-310 phosphodeficient and phosphomimic mutants (Mcm3^T310A^ and Mcm3^T310D^) was cloned into the pLew100-3HA vector. The resulting plasmids were each transfected in the 29–13 strain by electroporation. Transfectants were selected 2.5 μg/ml phleomycin and cloned by limited dilution as described above. Cells were incubated with 1.0 μg/ml tetracycline to induce the overexpression of 3HA-tagged Mcm3 and its mutants.

### In situ *epitope tagging of proteins*

Endogenous epitope tagging of proteins was performed using the one-step PCR-based epitope tagging method [62]. CRK2 and KKT13 were each tagged with a C-terminal triple HA epitope in the pSL-CRK2 cell line (both proteins) and the pSL-CYC13 cell line (KKT13 only), and transfectants were selected with 1 μg/ml puromycin and then cloned by limiting dilution. CYC13 was tagged with a C-terminal PTP epitope in the pSL-CYC13 cell line, and transfectants were selected with 10 μg/ml blasticidin and then cloned by limiting dilution.

For co-immunoprecipitation between CYC13 and CRK2, CRK3, or CRK12, CYC13 was tagged with a C-terminal PTP epitope in the Lister427 stain, and transfectants were selected with 40 μg/ml G418 and cloned by limiting dilution. Subsequently, the clonal cell line was used for tagging of CRK2, CRK3, or CRK12 with a C-terminal triple HA epitope, and transfectants were selected with 1 μg/ml puromycin and then cloned by limiting dilution.

For co-immunoprecipitation between Mcm3 and Cdc45 and between Mcm3 and Sld5, Mcm3 was tagged with a C-terminal triple HA epitope in the pSL-CRK2 RNAi cell line and the pSL-CYC13 cell line, and transfectants were selected with 1 μg/ml puromycin and cloned by limiting dilution. Subsequently, the clonal cell lines were used for tagging of Mcm2 or Cdc45 with a C-terminal PTP epitope or Sld5 with an N-terminal PTP epitope, and transfectants were selected with 10 μg/ml blasticidin and then cloned by limiting dilution.

For co-immunoprecipitation of Sld5 and wild-type and mutant Mcm3, Sld5 was endogenously tagged with an N-terminal PTP tag in the cells harboring pLew100-Mcm3-3HA, pLew100-Mcm3^T310A^-3HA, or pLew100-Mcm3^T310D^-3HA. Transfectants were selected with 1 μg/ml puromycin and cloned by limiting dilution. Expression of Mcm3-3HA, Mcm3^T310A^-3HA, and Mcm3^T310D^-3HA was induced with 0.1 μg/ml tetracycline for 6 h, and then harvested for cell lysis and immunoprecipitation.

### Flow cytometry

Flow cytometry was performed according to published procedures [43]. Cells were collected by centrifugation, washed with PBS, and then fixed with ethanol for 30 min at room temperature. Fixed cells were collected by centrifugation, washed once with PBS, and then suspended in PBS containing 10 μg/ml DNase-free RNase and 20 μg/ml propidium iodide. Cells were analyzed with a FACScan analytical flow cytometer (BD Biosciences) to quantitate the DNA content. The percentage of cells in different stages of the cell cycle was calculated by using the Kaluza Analysis software.

### In vitro *GST pull-down*

The full-length coding sequences of Mcm3 and Sld5 were each cloned into the pGEX-4 T-3 vector (Clontech), and the resulting plasmids were transformed into the *E. coli* BL21 strain. Expression GST-fusion proteins was induced with 0.2 mM IPTG for 4 h at room temperature and purified through binding to the Glutathione Sepharose 4B beads (GE HealthCare) according to the manufacturer’s instructions. GST-Mcm3 and GST-Sld5 bound on the Glutathione Sepharose beads were incubated with cleared lysate of *T. brucei* cells expressing CRK2-3HA at room temperature for 30 min. *T. brucei* cell lysate was prepared by lysing 10^8^ cells with 1 ml cell lysis buffer (25 mM Tris-Cl, pH 7.6, 100 mM NaCl, 1 mM DTT, 1% NP-40, and protease inhibitor cocktail) on ice for 30 min and then removing the insoluble pellets by centrifugation. 900 μl of cell lysate was used for pull-down, and the remaining 100 μl of cell lysate was set aside as the input sample. Beads were washed five times with the cell lysis buffer, and proteins were eluted by boiling the beads in 35 μl of 1x SDS-PAGE sampling buffer for 5 min. Eluted proteins (25 μl) and the input sample (15 μl) were fractionated by SDS-PAGE, transferred onto a PVDF membrane, and immunoblotted with anti-HA antibody. Purified GST was used as the negative control.

### In vitro *kinase assays*

The full-length coding sequences of CRK2 and CYC13 were each cloned into the pGEX-4 T-3 vector, and the resulting plasmids were transformed into *E. coli* BL21 strain. Recombinant GST-fused CRK2, CYC13, Mcm3, and Sld5 proteins were purified as described above. In vitro kinase assay was carried out using the method developed by the Kevin Shokat group, which uses a semisynthetic epitope for detection of thiophosphorylated kinase substrates [31]. In a 30-μl reaction setup, 10 μg purified GST-Mcm3, GST-Sld5, GST-CRK2, and GST-CYC13 were mixed in the kinase assay buffer (10 mM HEPES, pH 7.6, 50 mM NaCl, and 10 mM MgCl_2_) in the presence of 1 mM ATP-γ-S for 30 min at room temperature. Subsequently, 1.5 μl of 50 mM alkylating agent p-nitrobenzylmesylate (PNBM) was added into the kinase assay solution and incubated for 60 min at room temperature. Reaction was stopped by adding 1x SDS sampling buffer, and samples were separated by SDS-PAGE, transferred onto a PVDF membrane, and blotted with the monoclonal anti-ThioP antibody, which recognizes the thiophosphate ester (1:5000 dilution, ThermoFisher). GST-fusion proteins on the membrane were stained with coomassie brilliant blue.

For mass spectrometry analysis of potentially phosphorylated GST-Mcm3 and GST-Sld5, in vitro kinase assay was performed using 1 mM ATP and no PNBM was added to the reaction. GST-fusion proteins were separated on SDS-PAGE and stained with coomassie brilliant blue. The protein band corresponding to GST-Mcm3 and GST-Sld5 was excised from the SDS-PAGE gel and analyzed by LC-MS/MS to identify phospho-peptides.

### In-gel tryptic digestion and LC-MS/MS analysis of protein band

In-gel digestion of proteins was carried out as described previously [61]. Protein band was digested with trypsin (160 ng, Sigma-Aldrich) at 37 °C for 4 h, and peptides were extracted with 50 ml of 50% acetonitrile, 5% formic acid. Extracted peptides were dried using SpeedVac, reconstituted in 2% acetonitrile with 0.1% formic acid, and then injected onto Thermo LTQ Orbitrap XL (Thermo-Fisher Scientific, Bremen, Germany). Samples in 2% (v/v) acetonitrile and 0.1% (v/v) formic acid were analyzed on an LTQ Orbitrap XL (Thermo-Fisher Scientific) interfaced with an Eksigent nano-LC 2D plus ChipLC system (Eksigent Technologies, Dublin, CA). Sample was loaded onto a ChromXP C18-CL trap column (200 mm i.d. × 0.5 mm length) at a flow rate of 3 ml/min. Reversed-phase C18 chromatographic separation of peptides was carried on a on a ChromXP C18-CL column (75 mm i.d × 10 cm length) at 300 nl/min. The LTQ Orbitrap was operated in the data-dependent mode to simultaneously measure full-scan MS spectra in the Orbitrap and the five most intense ions in the LTQ by CID, respectively. In each cycle, MS1 was acquired at target value 1E6 with resolution R 1/4 100,000 (m/z 400) followed by top five MS2 scan at target value 3E4. The mass spectrometric setting is as follows: spray voltage was 1.6 KV, charge state screening and rejection of singly charged ion were enabled. Ion selection thresholds were 8000 for MS2, 35% normalized collision energy, activation Q was 0.25 and dynamic exclusion was employed for 30 s. Raw data files were processed and searched against the *T. brucei* database using the Mascot search engine. The search conditions used peptide tolerance of 10 p.p.m. and MS/MS tolerance of 0.8 Da with the enzyme set as trypsin and two missed cleavages permitted.

### Mcm3 RNAi by targeting the 3′UTR and functional complementation

To generate the Mcm3-3′UTR RNAi cell line, a 497-bp DNA fragment from the 3′UTR of Mcm3 gene, which does not overlap with the downstream gene, was amplified by PCR and cloned into the pZJM vector [63]. The resulting plasmid, pZJM-Mcm3-3′UTR, was linearized by NotI digestion and transfected into the 29–13 strain by electroporation. Trasfectants were selected with 2.5 μg/ml phleomycin and cloned by limited dilution as described above. To monitor the efficiency of Mcm3-3′UTR RNAi, Mcm3 was tagged with an N-terminal PTP epitope at one of its endogenous loci using PCR-based epitope tagging approach [62]. Transfectants were selected with 1 μg/ml puromycin, and cloned by limiting dilution as described above.

To generate the Mcm3 RNAi complementation cell lines, a 1920-bp DNA fragment (nucleotides 517–2436) of the coding sequence of Mcm3, Mcm3^T310A^, and Mcm3^T310D^ was cloned into the pC-3HA-BSD vector, which contains the 3′UTR from *TbRPA1* gene. The resulting plasmids were each linearized by restriction digestion with *Xcm*I and electroporated into the cells containing the pZJM-Mcm3-3′UTR construct and expressing PTP-Mcm3 from one of its endogenous loci (see above). Transfectants were selected with 10 μg/ml blasticidin and then cloned by limited dilution as described above. Clonal cell lines were verified by sequencing of the PCR product amplified using the forward primer targeting Mcm3 sequence and the reverse primer targeting TbRPA1 3′UTR sequence, and by western blotting with anti-HA antibody to detect 3HA-tagged Mcm3 and its mutants expressed from one Mcm3 locus and with anti-protein A antibody to detect PTP-tagged Mcm3 expressed from the other Mcm3 locus (see Fig. 5a, b for details).

### Co-immunoprecipitation

Co-immunoprecipitation was performed as described previously [64]. Briefly, 10^8^ cells were lysed in 1.0 ml cell lysis buffer (25 mM Tris-Cl, pH 7.6, 100 mM NaCl, 1 mM DTT, 1% NP-40, and protease inhibitor cocktail) for 30 min on ice. Cell lysate was cleared by centrifugation, and cleared cell lysate (0.9 ml) was incubated with 20 μl IgG Sepharose 6 fast flow beads (GE Healthcare) for 1 h at 4 °C. The remaining 100 μl of cell lysate was set aside as the input sample. Proteins bound to the IgG beads were eluted by boiling the beads in 35 μl of 1x SDS sampling buffer. Subsequently, 25 μl of the eluted proteins and 15 μl of the input sample were loaded onto a SDS-PAGE gel for protein separation. Proteins were transferred onto a PVDF membrane and immunoblotted with anti-HA antibody and anti-protein A antibody (Sigma-Aldrich). Intensity of the protein band was measured with the ImageJ software.

### EdU incorporation assay

The Click-iT™ EdU Alexa Fluor 488 imaging kit (ThermoFisher Scientific) was used to detect the incorporation of EdU into DNA. Cells were cultured in SDM-79 medium supplemented with 30 mM EdU for 3 h. Cells were collected by centrifugation, washed with PBS, adhered to glass coverslips, and fixed with 4% paraformaldehyde. Cells on the coverslips were used for EdU detection according to the manufacturer’s instructions. Cells were visualized under a fluorescence microscope, and EdU-positive cells were counted.

### Immunofluorescence microscopy

Cells were washed once with PBS, settled onto glass coverslips for 30 min at room temperature, and fixed with cold methanol for 30 min at − 20 °C. Cells were rehydrated with PBS and incubated with the blocking buffer (3% BSA in PBS) for 1 h at room temperature. Cells were incubated with the FITC-conjugated anti-HA monoclonal antibody (1:400 dilution, Sigma Aldrich, Clone HA7) for 1 h at room temperature and washed three times with PBS. Coverslips were washed three times with PBS and mounted with DAPI-containing VectaShield mounting medium (Vector Labs). Immunostained cells were imaged with an inverted fluorescence microscope (Olympus IX71) equipped with a cooled CCD camera (model Orca-ER, Hamamatsu) and a PlanApo N 60 × 1.42-NA lens. Images were acquired using the Slidebook5 software and processed using the Adobe Photoshop software.

### Statistical analysis

Statistical analysis was performed using the chi-square test in the Microsoft *Excel* software. Detailed *n* values for each panel in the figures were stated in the corresponding figure legends. For immunofluorescence microscopy, images were randomly taken, and all cells in the image were counted.

## Supplementary Information

**Additional file 1: Figure S1.** Sequence alignment of the twelve cyclin proteins (CYC2-CYC13) in *T. brucei*. **Figure S2.** Co-immunoprecipitation to test the in vivo interaction between CYC13 and CRK3 and between CYC13 and CRK12. **Figure S3.** Quantification of KKT13 fluorescence intensity in non-induced control, CRK2 RNAi and CYC13 KPP1 RNAi cells. **Figure S4.** Coverage of the peptides detected by mass spectrometry in purified GST-Sld5 and GST-Mcm3 after in vitro kinase assay with CRK2-CYC13. **Figure S5.** Effect of epitope tagging of Mcm3 on its function and cell growth. **Figure S6.** Ectopic overexpression of Mcm3 and its mutant in the 29–13 strain.

**Additional file 2: Figure S7.** Full western blots used for Fig. 1e, f. **Figure S8.** Full western blots used for Fig. 2. **Figure S9.** Full western blots used for Fig. 4b, c. **Figure S10.** Full western blots used for Fig. 5c. **Figure S11.** Full western blots used for Fig. 6a-e. **Figure S12.** Full western blots used for Figure S2. **Figure S13.** Full western blots used for Figure S5. **Figure S14.** Full western blots used for Figure S6.

## Data Availability

All data reported in this paper are included in this published article and the supplementary information files. Full western blots were included as Additional file 2. All materials and cell lines reported in this paper are available upon request.
