# Supplementary material for: The CRK2-CYC13 complex functions as an S-phase cyclin-dependent kinase to promote DNA replication in Trypanosoma brucei
Source: BMC Biol. 2021 Feb 11;19:29. doi: 10.1186/s12915-021-00961-1 (PMC7876812; doi:10.1186/s12915-021-00961-1)
Supplement: Supplementary file 1 — Additional file 1: Figure S1. Sequence alignment of the twelve cyclin proteins (CYC2-CYC13) in T. brucei. Figure S2. Co-immunoprecipitation to test the in vivo interaction between CYC13 and CRK3 and between CYC13 and CRK12. Figure S3. Quantification of KKT13 fluorescence intensity in non-induced control, CRK2 RNAi and CYC13 KPP1 RNAi cells. Figure S4. Coverage of the peptides detected by mass spectrometry in purified GST-Sld5 and GST-Mcm3 after in vitro kinase assay with CRK2-CYC13. Figure S5. Effect of epitope tagging of Mcm3 on its function and cell growth. Figure S6. Ectopic overexpression of Mcm3 and its mutant in the 29–13 strain. [file 12915_2021_961_MOESM1_ESM.pdf]

Figure S1

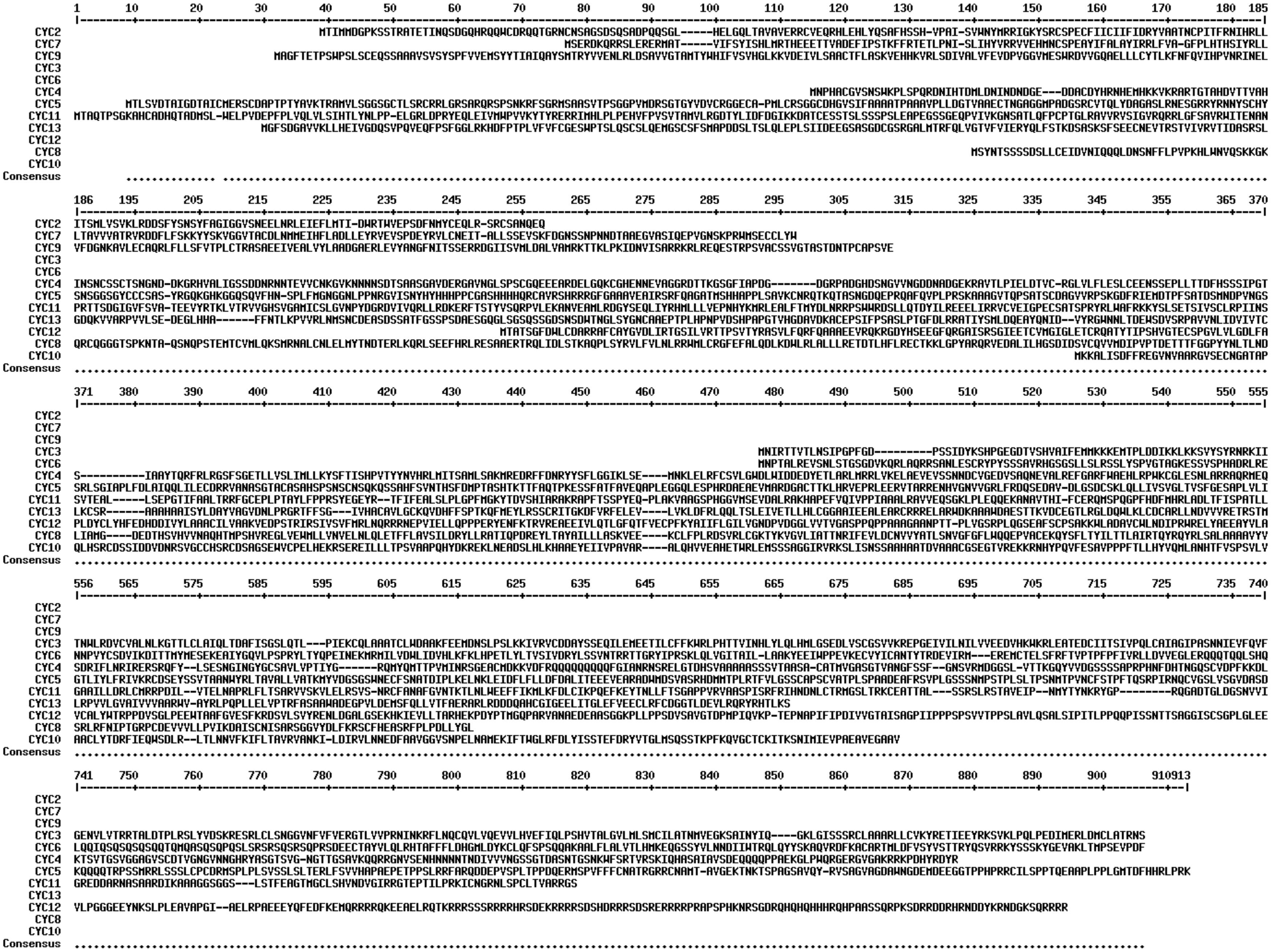

Figure S1. Sequence alignment of the twelve cyclin proteins (CYC2-CYC13) in *T. brucei*.

Figure S2

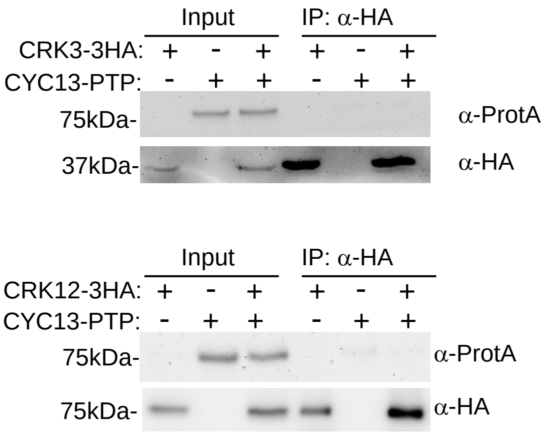

Figure S2. Co-immunoprecipitation to test the *in vivo* interaction between CYC13 and CRK3 and between CYC13 and CRK12. CYC13 was endogenously tagged with a PTP epitope, whereas CRK3 and CRK12 were endogenously tagged with a triple HA epitope. CYC13-PTP alone and 3HA-tagged CRK3 and CRK12 alone were included as negative controls. IP was performed with anti-HA agarose to pull down 3HA-tagged CRK3 and CRK12. Immunoprecipitated proteins were immunoblotted with anti-HA antibody (α-HA) to detect CRK3-3HA and CRK12-3HA and with anti-Protein A antibody (α-ProtA) to detect PTP-CYC13.

Figure S3

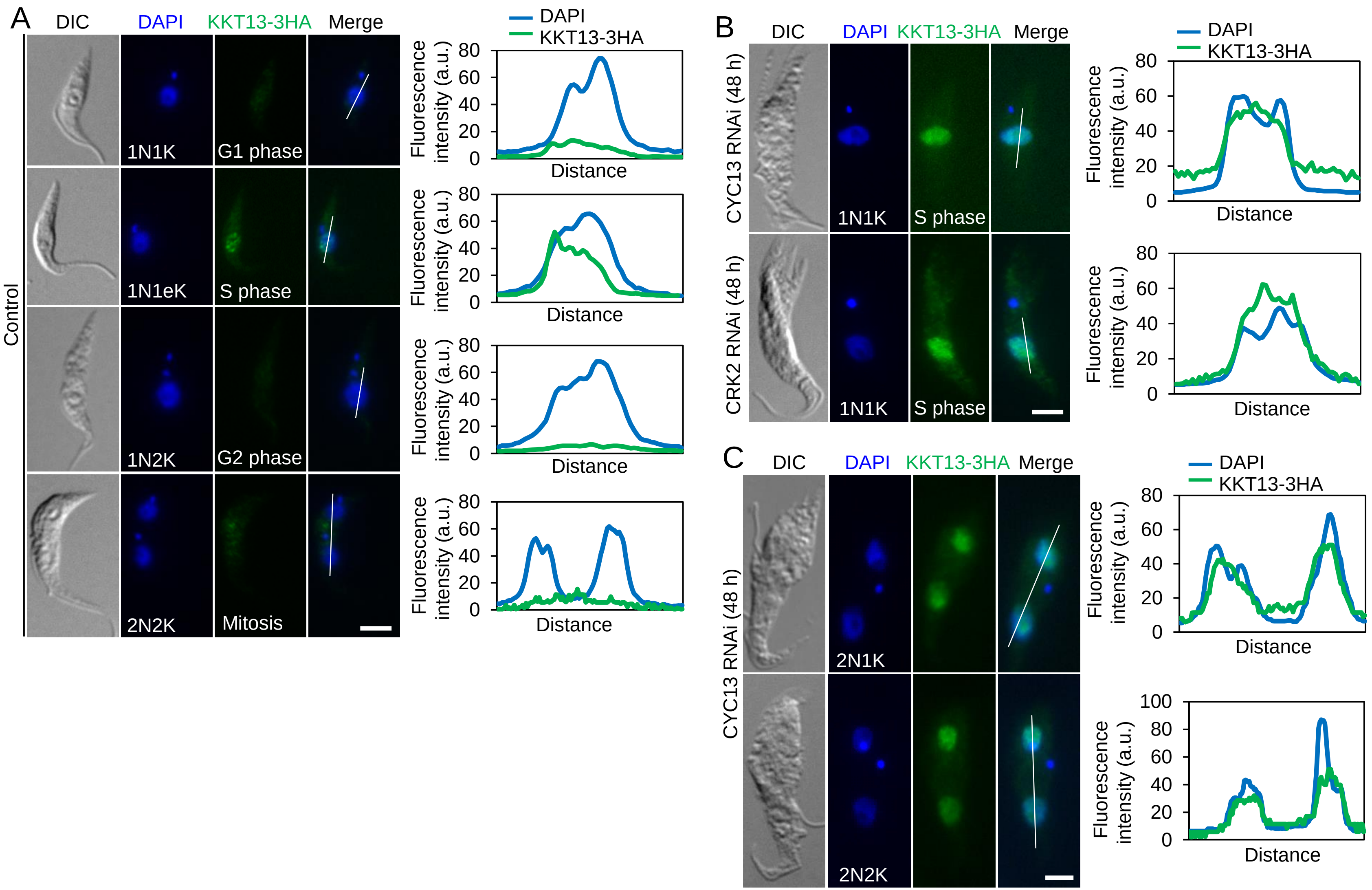

Figure S3. Quantification of KKT13 fluorescence intensity in non-induced control, CRK2 RNAi and CYC13 KPP1 RNAi cells. Cells were immunostained with FITC-conjugated anti-HA antibody and counterstained with DAPI to label nucleus and kinetoplast. Shown on the right are histograms of the quantitation of the intensity of KKT13-3HA fluorescence signal (green) and DAPI signal (blue) across the white line in the merged image on the left. Images are representative of n = 3 independent experiments. (**A**). Non-induced control cells at different cell cycle stages. (**B**). CYC13 RNAi and CRK2 RNAi cells of the 1N1K configuration. (**C**). CYC13 RNAi cells of the 2N1K and 2N2K configuration. Scale bar: 5 µm.

Figure S4

Sld5 (1097 peptides, 16 unique peptides, 76.2% coverage)

MFFEDQLDEALSREGSPRLSTSNTVGGADLVAAGAANDETFPFPSHAAPGHLLKSLIQATENERC
APDILPYPEAIVDSVVAQIVAQNEQIRLLGTDERQKAAGSDSGVSLLPFKPSDIMALEVQRAQFF
LCELLRCRLRKIEALALTINYESQSGAEAHTQLREHLSHNEIVVADRLAELISKCVRQAGLQSAP
SELQQLVPNAPYAEGNEVLPIPDIDHYVFCVVLDDLGVVRLGDDAEQTVHAGEVFIVPYRTFRPY
ILEGRVRLV

Mcm3 (3997 peptides , 91 unique peptides, 87.9% coverage)

MLSNRTLTEEQTLIRRHFIDFFESERYEEKYHQLVQEMMAASGSRLLLDMGDLLDFTPTATGFDT
TAGLGLNQSASLGACIIREPGKYVPLLELALHDVVLRQQPEYLKVDYRSRAVHVGFEGPVGTVRS
PRELYAQHLNTMVALEGIITRQSSNRPRVLETVHYCPETNKFSRKEFRDQLTPMIDSRHLPTVNV
MPKTDMEGNVLRTELGLSTFMDSQCAILQEAPERAPTGQLPRTVELRFDDDLVDIVKPGDRVTLV
GVYMAYTTSDNKSFQSIVLVNHVIPVQAFTMYRRVPSIEEKLFAFAAKQTQTDGPAGVLNSLSMA
VAPTIYGMTNERKAVLLLMVGGVERVAHQSHVRGDINVLLVGEPSTAKSQLLRFVLGVAPLALST
TGKGSSGVGLTAAVSIDAYTGERSLSAGAMVLADRGILCIDEFDKMSAQDRVAMHEAMEQQTVTI
AKAGIHASLNARCSVLAAANPIYGFYSVHHRLAFNVGLPESLLSRFDLTFIVLDKHSSEHNRRIG
RHILRNHMTAEPVEIDQNITKTVVDSVDSVWAQSGRQGGGAGGGLDFRMTTTSSGETIVGVDFLR
AYVQLAKLGRPTLTEASQQLVSQHYVQLRAEQQEGSKDGFFVTARTLEAIVRLATANAKLRLSST
VDEDDVKSAMELLRASVHAATAASQQRAEDNKATAMEKRQGAKRSAEGLTNGAGVDIISGAGNTR
RQRQEVEETAAGAVASPAEEMALTTAESSKEITLSRVSNMLRLIQRQDRQPAVRLSDVHARLGGS
LSMEELRQAVSELQGDSFVYEAIGEDEWVQFI

Figure S4. Coverage of the peptides detected by mass spectrometry in purified GST-Sld5 and GST-Mcm3 after in vitro kinase assay with CRK2-CYC13. Peptides detected by mass spectrometry are in red. The peptides containing the phosphorylated Serine or Threonine residues are underlined, and the residues outlined in green boxes are phosphoryated residues in Mcm3 (Ser-213 and Thr-310).

Figure S5

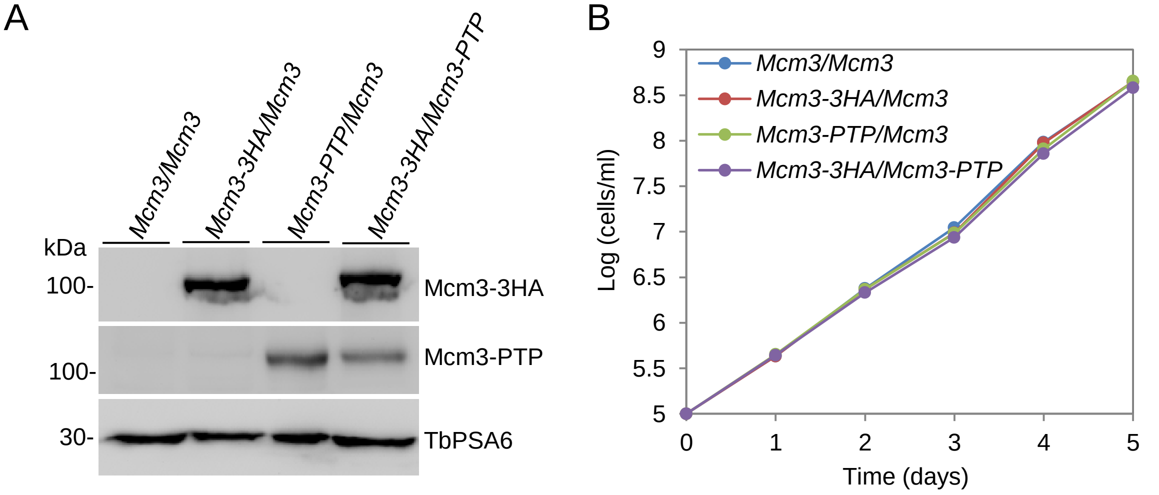

Figure S5. Effect of epitope tagging of Mcm3 on its function and cell growth. (**A**). Western blotting to detect the endogenous 3HA-tagged and/or PTP-tagged Mcm3. Mcm3-3HA was detected by anti-HA antibody, and Mcm3-PTP was detected by anti-Protein A antibody. TbPSA6 was detected by anti-TbPSA6 antibody and served as the loading control. Shown are the wild-type strain (*Mcm3/Mcm3*), a cell line with Mcm3 tagged with a triple HA from one of the two loci (*Mcm3-3HA/Mcm3*), a cell line with Mcm3 tagged with a PTP epitope from one of the two loci (*Mcm3-PTP/Mcm3*), and cell line with Mcm3 tagged with a triple HA from one locus and with a PTP epitope from another locus (*Mcm3-3HA/Mcm3-PTP*). (**B**). Growth curves of the 427 strains with different genotypes (*Mcm3-3HA/Mcm3*, *Mcm3-3HA/Mcm3, Mcm3-PTP/Mcm3, and Mcm3-3HA/Mcm3-PTP*).

Figure S6

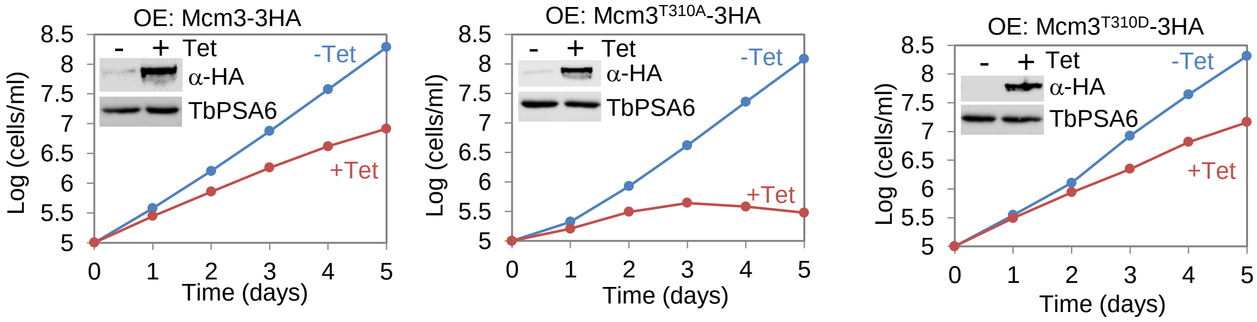

Figure S6. Ectopic overexpression of Mcm3 and its mutant in 29-13 strain. Ectopic overexpression of 3HA-tagged wild-type, Thr-310 phospho-deficient mutant, and Thr-310 phospho-mimic mutant Mcm3 in the 29-13 strain. Cells were induced without (-Tet) or with (+Tet) 1.0 µg/ml tetracycline for 5 days and cell growth was monitored by daily counting of cells. The insets showed the Western blots to monitor the expression of 3HA-tagged wild-type and mutant Mcm3 with anti-HA antibody. TbPSA6 was detected by anti-TbPSA6 antibody and served as the loading control.
