# Supplementary material for: The CRK2-CYC13 complex functions as an S-phase cyclin-dependent kinase to promote DNA replication in Trypanosoma brucei
Source: BMC Biol. 2021 Feb 11;19:29. doi: 10.1186/s12915-021-00961-1 (PMC7876812; doi:10.1186/s12915-021-00961-1)
Supplement: Supplementary file 2 — Additional file 2: Figure S7. Full western blots used for Fig. 1e, f. Figure S8. Full western blots used for Fig. 2. Figure S9. Full western blots used for Fig. 4b, c. Figure S10. Full western blots used for Fig. 5c. Figure S11. Full western blots used for Fig. 6a-e. Figure S12. Full western blots used for Figure S2. Figure S13. Full western blots used for Figure S5. Figure S14. Full western blots used for Figure S6. [file 12915_2021_961_MOESM2_ESM.pdf]

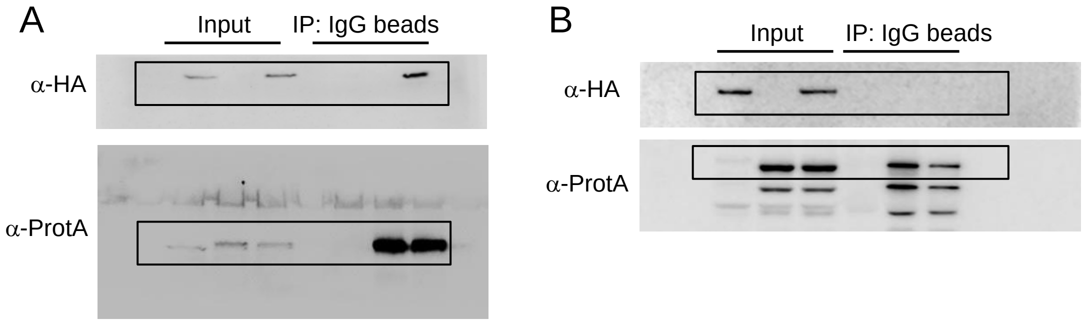

FigS7. Full western blots used for Fig.1E, F. (A). Full western blots used for Fig.1E. (B). Full western blots used for Fig.1F.

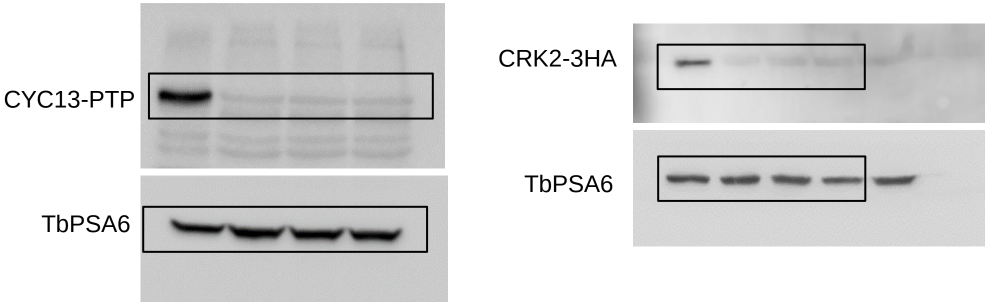

Fig S8. Full western blots used for Fig.2A

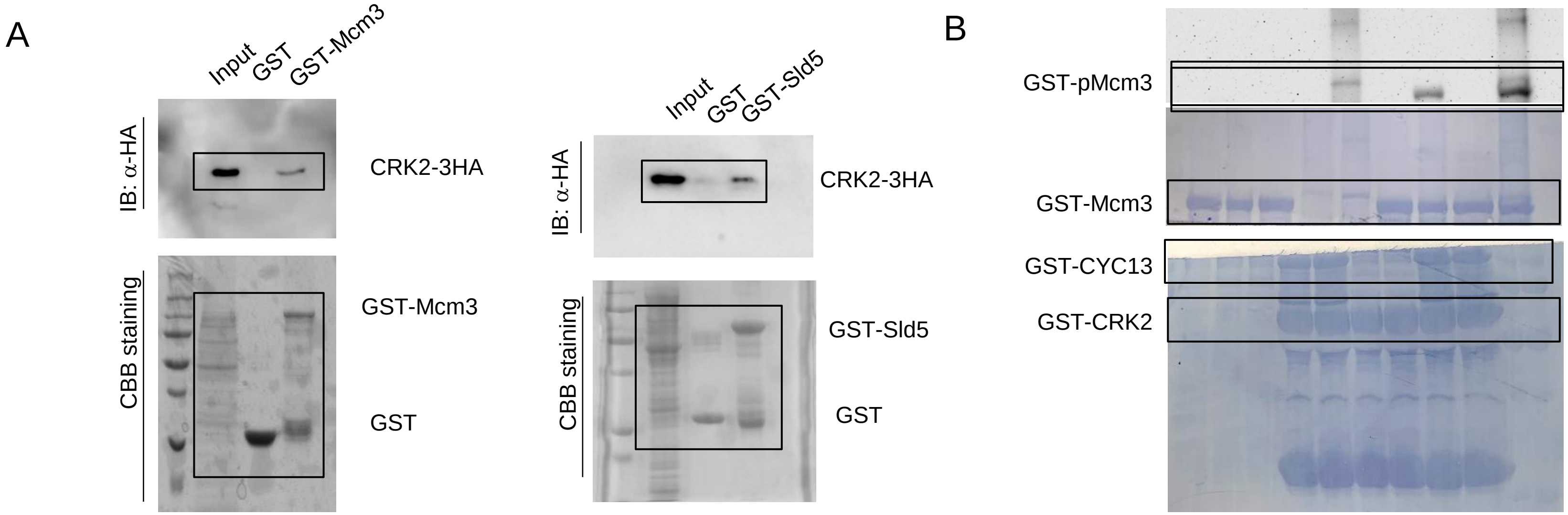

FigS9. Full western blots used for Fig.4B, C . (A). Full western blots used for Fig.4B. (B). Full western blots used for Fig.4C.

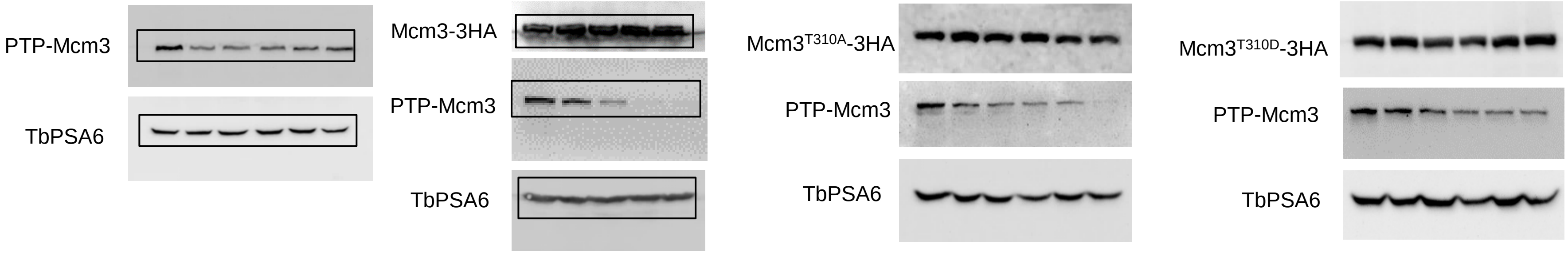

FigS10. Full western blots used for Fig. 5C.

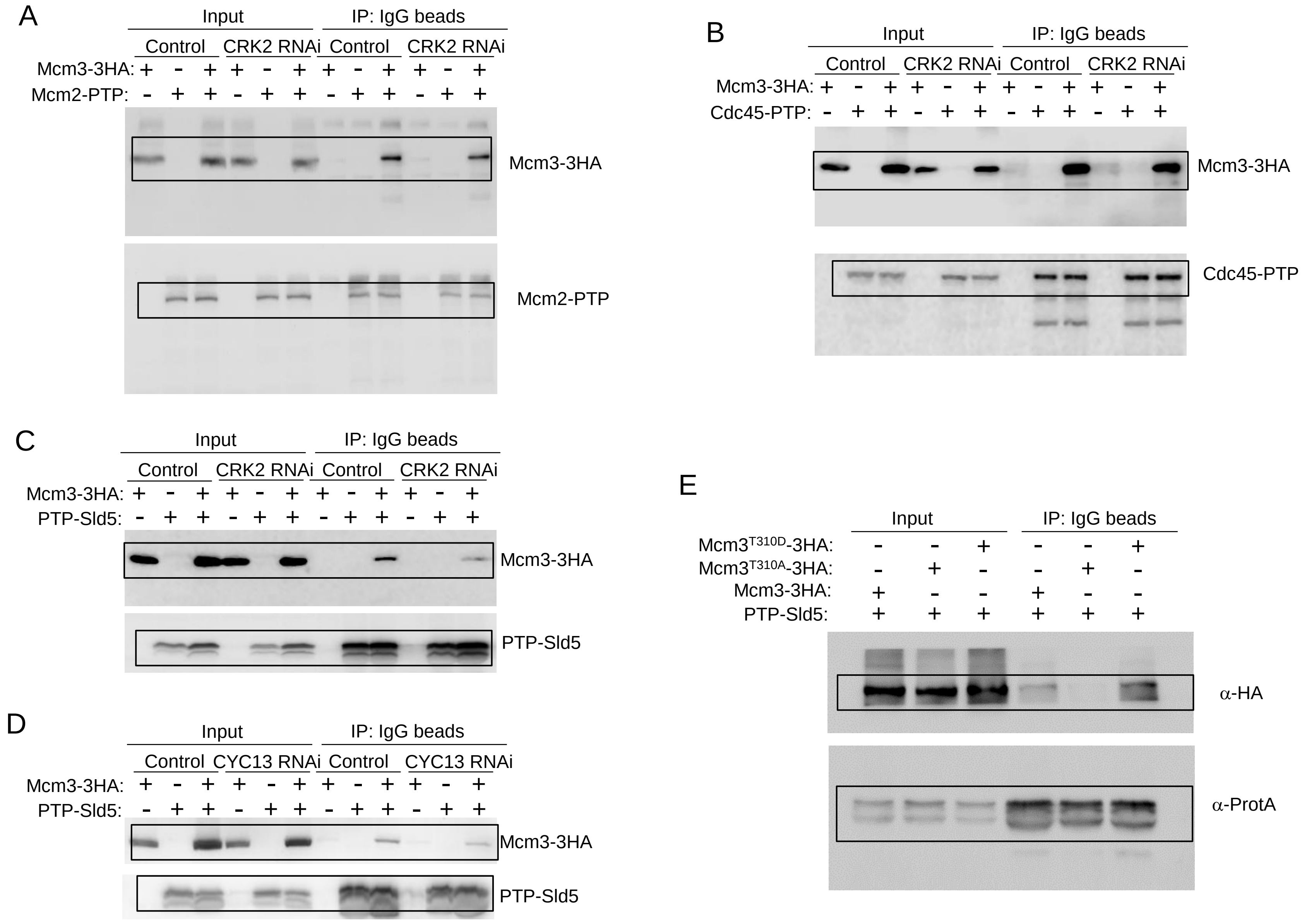

FigS11. Full western blots used for Fig.6 A,B,C,D ,E. (A). Full western blots used for Fig.6A. (B). Full western blots used for Fig.6B. (C). Full western blots used for Fig.6C. (D). Full western blots used for Fig.6D. (E). Full western blots used for Fig.6E.

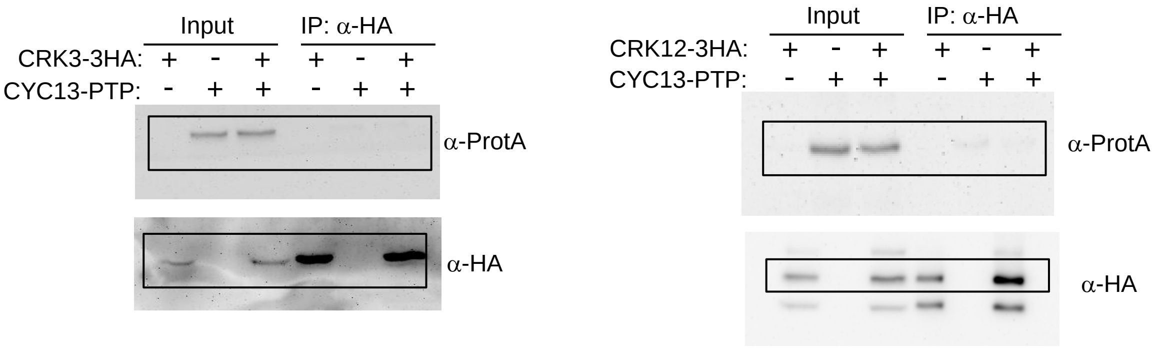

FigS12. Full western blots used for Fig. S2

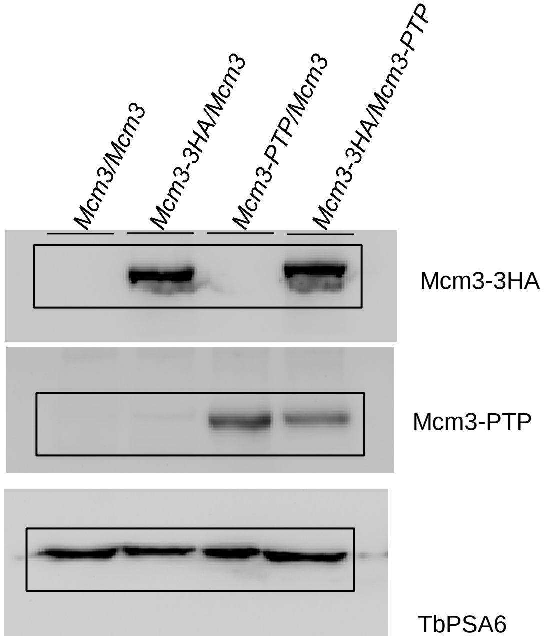

Fig S13. Full western blots used for Fig. S5

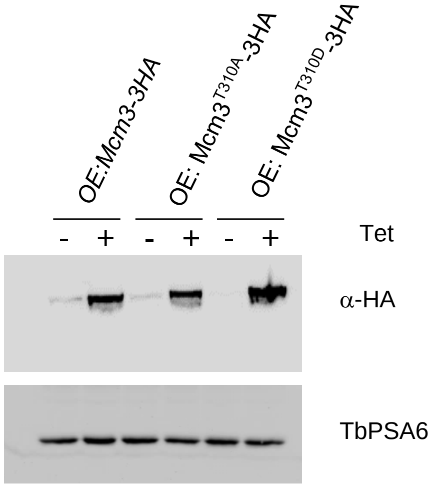

Fig S14. Full western blots used for Fig. S6
